# Intracranial Calcifications in Systemic Lupus Erythematosus

**DOI:** 10.7759/cureus.27952

**Published:** 2022-08-12

**Authors:** Arshia Bhardwaj, Tulika Garg, Monica Gupta, Narinder Kaur, Samiksha Gupta

**Affiliations:** 1 General Medicine, Government Medical College & Hospital, Chandigarh, IND; 2 Medicine, Government Medical College & Hospital, Chandigarh, IND; 3 Radiodiagnosis, Government Medical College & Hospital, Chandigarh, IND

**Keywords:** calcification, neurological manifestation, central nervous system, intracranial calcifications, systemic lupus erythematosus

## Abstract

We present an unusual case of a 37-year-old woman diagnosed with systemic lupus erythematosus presenting with right-sided weakness and altered mentation. On computed tomography and magnetic resonance imaging, marked intracranial calcifications were seen. These localized calcifications are speculated to be secondary to the necrotic focus of repeated episodes of vessel inflammation. However, the pathogenesis of cerebral calcifications is largely unknown.

## Introduction

Neurological involvement in systemic lupus erythematosus (SLE) may present either as central nervous system (CNS) or neuropsychiatric symptoms such as cognitive impairment, psychosis, depression, stroke, seizure, movement disorder, or peripheral neuropathy [1]. The most commonly found abnormalities in neuroimaging are cerebral atrophy, infarction, or intracerebral hamartomas [2]. Calcifications of the cortex, basal ganglia, and cerebellum have been reported in very few cases, making it an unusual presentation in SLE.

The underlying mechanism of these calcifications in SLE is unknown but may be dystrophic following microinfarctions due to primary vascular damage and ongoing venous inflammation. In the brain, 5.6% of cases had vascular involvement while histopathological findings were suggestive of non-inflammatory vasculopathy with secondary infarcts [3]. The basal ganglia are the most common site for localized calcifications, with lesser involvement of other regions such as the thalamus and cerebellum [4].

## Case presentation

A 37-year-old woman was admitted to the hospital with complaints of fever, right-sided weakness of two weeks duration, inability to swallow, and altered confusional state for the past three days. She also complained of facial rash which was exacerbated by sun exposure, hair loss, and inflammatory small joint pains. On examination, she had a typical butterfly-shaped malar rash and non-scarring alopecia. The neurological examination revealed positive Babinski sign, rigidity, and hyperreflexia in both right upper and lower limb (power of 3/5) while the power on the left side was 5/5 according to the medical research council (MRC) scale. She also had gait ataxia with dysarthria. However, there were no meningeal signs or peripheral neuropathy. She had no history of bluish discoloration of fingers, skin tightening, dryness of mouth and eyes, seizures, or psychosis. There was also no history of prior CNS infections or any cranial irradiation. The patient was diagnosed with SLE, six months prior to the current presentation based on the Systemic Lupus International Collaborating Clinics (SLICC 2012) criteria (three clinical and one immunological). Hence, she was taking 5 mg of prednisolone along with 200 mg of hydroxychloroquine daily for SLE.

Laboratory investigations (Table 1) revealed a high titer for antinuclear antibodies (1:320 dilution by enzyme-linked immunosorbent assay (ELISA) and speckled pattern on indirect immunofluorescence test). The anti-double-stranded deoxyribonucleic acid (anti-dsDNA) titers by ELISA were, however, intermediate (65 IU/mL; Normal range: <30 IU/mL). Complement levels (C3: 102 U/mL; normal: 80-120 and C4 38 mg/dl; normal: 15-45 mg/dL) were within normal limits, excluding lupus flare. The antiphospholipid (aPL), anticardiolipin (aCL) antibodies, and lupus anticoagulant (LA) were negative. The complete blood count, liver, kidney functions, and serum electrolytes were within normal limits. There was no evidence of proteinuria on urine analysis. She had normal blood calcium, phosphorus, and vitamin D levels. Thyroid, parathyroid function tests, and 24-hour urinary calcium levels were also within normal limits.

**Table 1:** Investigations done at the time of presentation ALP: alkaline phosphatase; GGT: gamma-glutamyl transferase; AST: aspartate transaminase; ALT: alanine transaminase; ESR: erythrocyte sedimentation rate; CRP: C-reactive protein; TSH: thyroid stimulating hormone; iPTH: parathyroid hormone; RA factor: rheumatoid factor; ANA: antinuclear antibody; Anti-dsDNA: Anti-double-stranded DNA antibody; C3: complement levels; C4: complement levels

Investigation	Value (Day 1)	Normal range
Hemoglobin (g/dL)	12.5	13-16
Platelets (×10^9^/L)	3.25	150-400
Total leukocyte count (×10^9^/L)	9.5	4-12
Bilirubin (mg/dL)	0.7	0.2-1
ALP (U/L)	46	30-150
GGT (U/L)	33	<50
AST (U/L)	35	10-40
ALT (U/L)	22	10-40
Albumin (gm/dL)	4.8	3.5-5.5
Globulin (gm/dL)	3.0	2-3.5
Sodium (mmol/L)	140	135-145
Potassium (mmol/L)	4.0	3.5-5.5
Urea (mg/dL)	22	15-40
Creatinine (mg/dL)	0.5	<1.3
ESR (mm/hr)	26	0-20
CRP (mg/L)	04	<5
Calcium (mg/dL)	9.2	8.0-10.4
Phosphorus (mg/dL)	2.0	2.5-4.5
T3 (ng/ml)	0.85	0.60-1.81
T4 (µg/ml)	8.9	3.2-12.6
TSH (µIU/mL)	2.57	0.35-5.50
iPTH (U/mL)	65	<80
Vitamin D (ng/mL)	45	30-100
RA factor (U/mL)	5.0	<10
ANA	1:320	<1:40
Anti- dsDNA (U/mL)	65	<30
C3 (U/mL)	102	80-120
C4(mg/dL)	38	15-45

Her serology for human immunodeficiency virus (HIV) was negative by ELISA. Ultrasound for thyroid and parathyroid glands revealed no abnormality. Ultrasound abdomen was also grossly normal with no visceral calcifications, normal kidney size, and echotexture. The Systemic Lupus Erythematosus Disease Activity Index 2000 (SLEDAI-2K) was 24 [5]. A cerebrospinal fluid (CSF) analysis was done to rule out CNS infection (acellular with glucose of 65 mg/dl, protein of 48 mg/dl) and CSF culture was sterile and negative for herpes simplex virus polymerase chain reaction (HSV PCR) and India ink preparation. Electroencephalography (EEG) revealed generalized cerebral dysfunction.

**Figure 1:**
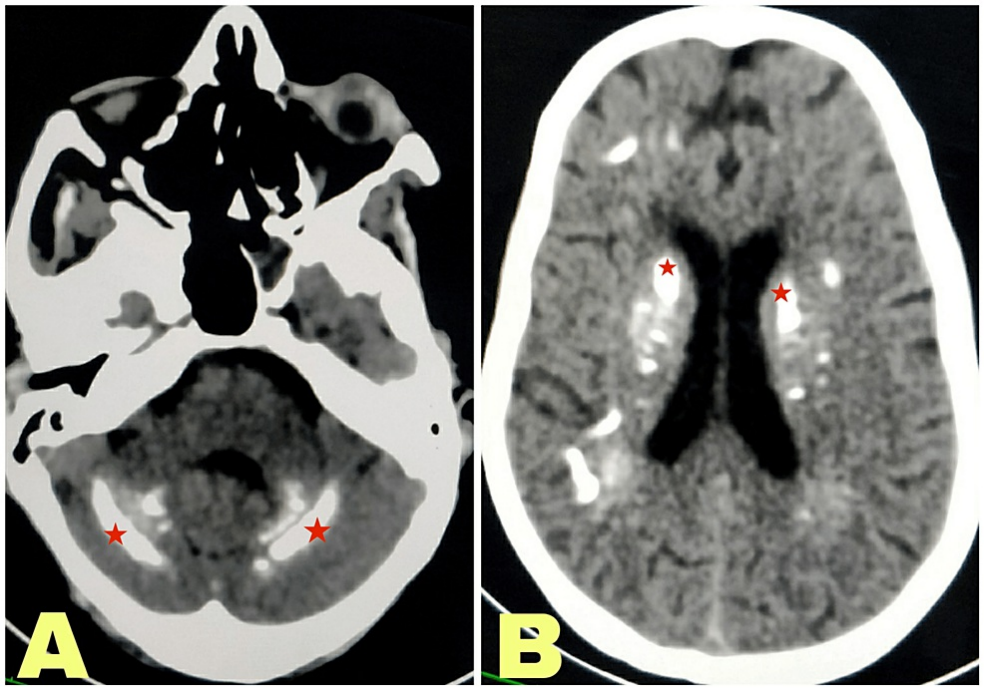
(A) Axial non-contrast CT scan at the level of cerebellum showing bilateral calcification in the region of cerebellar cortex (red star); (B) Axial non-contrast CT scan at the level of basal ganglia demonstrating bilateral calcification in the region of caudate nucleus, globes pallidum, and putamen (red star)

**Figure 2:**
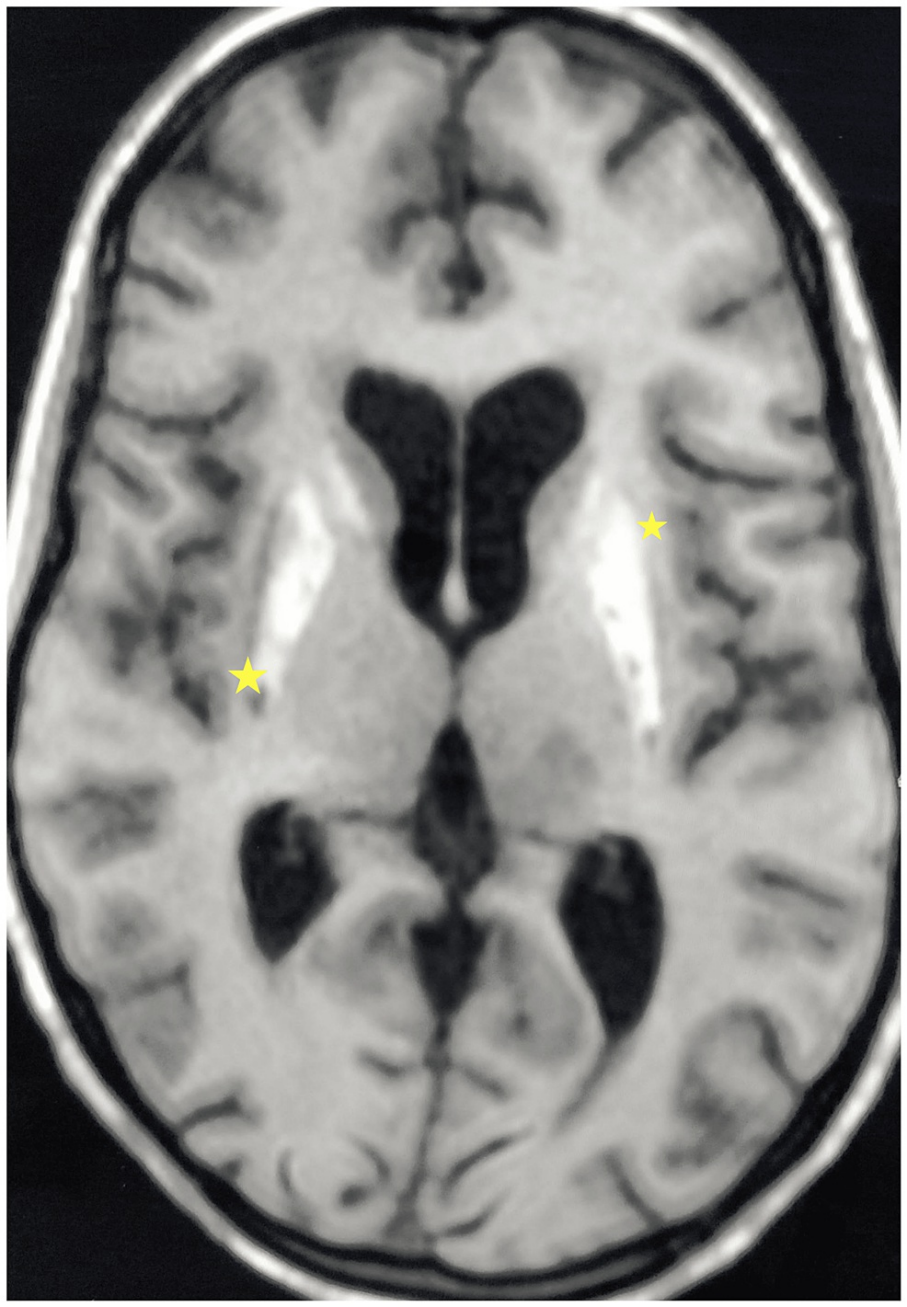
T1-weighted axial non-contrast MRI brain showing bilateral patchy hyperintense signals in bilateral basal ganglia (yellow star) regions suggestive of calcification

Plain computed tomography (CT) head revealed calcifications in subcortical white matter, bilateral corona radiata, periventricular region, bilateral basal ganglia, brain stem, dentate nuclei, and cerebellar folia (Figure 1). The same findings were confirmed in non-contrast magnetic resonance imaging (MRI) brain, which was suggestive of hyperintense basal ganglia and dentate nuclei on T1-weighted images (Figure 2). Similar blooming was seen in bilateral basal ganglia, dentate nuclei, cerebellar folia, and subcortical and juxtacortical white matter in bilateral cerebral hemispheres. In addition to this, MRI also showed multifocal subacute infarcts in the left thalamus, right internal capsule, anterior body of corpus callosum, periventricular white matter of left temporal lobe, left cerebellar hemisphere, and left cerebral peduncle. Considering the acute neurological event and her increased disease activity score, her dose of steroids was hiked up to 1 mg/kg/day. The patient had considerable improvement in the weakness of the right upper and lower limb (power 5/5) on day seven of follow-up.

## Discussion

SLE is a chronic autoimmune disorder with up to 75% predilection for the nervous system; however, these may not be recognized due to the diverse and varied presentations [6]. Bilateral symmetric intracranial calcification is known to occur in several conditions like hypoparathyroidism, Fahr’s disease, anoxic encephalopathy, and idiopathic ferrocalcinosis [7]. Certain hereditary diseases (Cockayne’s syndrome, Albright’s osteodystrophy, Down’s syndrome), intoxications (lead and carbon monoxide), and CNS infections (tuberculous meningitis, herpes, or measles encephalitis) have also been associated with basal ganglia calcification [8]. Our patient did not have any endocrine or electrolyte abnormalities or a family history of any of these diseases. Hence, the above observations suggest that these intracranial calcifications in our patient likely resulted from vasculopathy of SLE. Limited cases of CNS calcifications in SLE have been described in the literature.

For the diagnosis of calcification, CT and MRI brain imaging are both valuable radiological techniques. Irrespective of the etiology, calcified deposits display a uniform distribution and this may be attributed to the selective exposure of certain areas of the brain for the deposition of calcium. Since the calcium concentration seems to be higher in the basal ganglia compared to other areas of the brain, it is the most frequent site for localization [9]. Only a few cases of calcifications have been reported in the cerebellum, white matter, and cortex [10,11]. Matsumoto et al. reported a case of intracerebral calcifications and depicted that the calcifications were around the venous vessels in the core of the necrotic area. Their study suggested that the neurotoxic factors exuded from venous vessels result in calcifications [12]. Raymond et al. revealed the hypothesis of primary immunological vascular damage, which triggers microinfarctions with posterior dystrophic calcification [4].

Several SLE patients with calcium deposition have never had neurological manifestations, and no major disparities have been observed in the neurological picture in SLE patients with or without cerebral calcification. It can be a possibility that calcium deposition has no direct role in the clinical expression of CNS lupus. The pathogenesis of these findings is unclear and there is no confirmed hypothesis, but possibly the calcifications are a result of repeated episodes of venous inflammation with focal immunological demyelination. A probable mechanism can be the leakage of proteins from venous vessels with neurotoxic and pro-calcification properties.

## Conclusions

Massive intracranial calcification can rarely be seen in cases of SLE, but the association between the neurological events and calcification in the specific areas of brain could not be proven in this case or in any of the prior case reports. The mechanism of intracranial calcification in SLE remains unclear, but it should be borne in mind that marked intracranial calcification can be observed in various rheumatological disorders such as SLE, systemic sclerosis, or dermatomyositis.
